# The Role of Neoadjuvant Therapy in a Giant Gastric Gastrointestinal Stromal Tumour: A Case Report and Review of the Literature

**DOI:** 10.7759/cureus.55655

**Published:** 2024-03-06

**Authors:** Jolan S Alsaud, Saja Alruqayi, Abdulaziz Alomair

**Affiliations:** 1 College of Medicine, Qassim University, Buraidah, SAU; 2 Clinical Pharmacy, Qassim University, Buraidah, SAU; 3 Surgery, King Fahad Specialist Hospital, Buraidah, SAU

**Keywords:** the rule of neoadjuvant therapy, neoadjuvant therapy, gastric gastrointestinal stromal tumor, large gastric gastrointestinal stromal tumor, huge gastric gastrointestinal stromal tumor

## Abstract

Gastric gastrointestinal stromal tumour (GIST) is a rare disease with an annual incidence of 10 cases per million. Herein, we present the case of a 45-year-old man who visited our clinic with complaints of weight loss and anorexia, without changes in bowel habits or vomiting, for four months. On physical examination, all vital signs were normal. The abdomen was distended without tenderness and had a giant upper abdominal mass. Tumour marker investigation revealed high levels of cancer antigen 125 with normal levels of alpha-1-fetoprotein, carcinoembryonic antigen, and carbohydrate antigen. A computed tomography (CT) scan showed a mass measuring 35 × 25 × 20 cm, likely originating from the fundus of the stomach. Upper gastrointestinal endoscopy indicated external compression of the stomach and a fundal submucosal mass. Ultrasound-guided biopsy demonstrated the presence of a GIST. There was a severe danger of both the tumour rupturing during surgery and the combined excision of adjacent organs if the surgery was performed with the massive tumour. Therefore, daily neoadjuvant therapy with imatinib 400 mg was administered for three months. Post-therapeutic CT indicated a significant reduction in the size of the mass, which now measured 17 × 14 × 21 cm. The patient underwent surgical resection a month after the completion of neoadjuvant therapy, and the post-operative period was uneventful. He was followed up regularly at the general surgery department for 24 months without recurrence. This case asserts the benefit of neoadjuvant therapy in reducing the tumour size pre-operatively, which enhances the complete resection rate, prevents the need for multi-organ resection, and lowers the risk of surgery.

## Introduction

Although the most prevalent mesenchymal tumour of the gastrointestinal tract is the gastric gastrointestinal stromal tumour (GIST), it is a rare disease with an annual incidence of approximately 10 cases per million [1]. A majority of GISTs include an activating mutation in either platelet-derived growth factor receptor-α (PDGFRA; 5%-10%) or KIT proto-oncogene receptor tyrosine kinase (KIT; 75-80%) [2]. Less than half of the patients with GIST initially present with localized primary illness, and 40%-90% of those surgically treated experience post-operative recurrence or metastasis [3-5]. Improving the resection success rate is the most crucial aspect of extending the life of patients with GIST. In those with metastatic or incurable GIST, imatinib, a small-molecule inhibitor of the oncoproteins PDGFRA and KIT, has demonstrated efficacy in clinical trials [6,7]. Furthermore, imatinib has been shown to be beneficial as adjuvant treatment in randomized clinical trials for patients with a high risk of recurrence [8-10]. This drug improves the overall survival and recurrence-free survival of patients with GIST who have a high risk of relapse after surgery and is now considered the standard treatment [11]. It is anticipated that neoadjuvant therapy with imatinib will cause a notable decrease in tumour size, which might enhance the complete resection rate, prevent the need for multi-organ resection, and lower the risk of surgery. This report presents a case of giant gastric GIST. Additionally, it highlights the benefits of neoadjuvant therapy in reducing the tumour size before surgery, which enhances the complete resection rate, prevents the need for multi-organ resection, and lowers the risk of surgery.

## Case presentation

A 45-year-old man visited our clinic with complaints of weight loss and anorexia, without changes in bowel habits or vomiting, for four months. He had no history of medical disease or previous surgery. On physical examination, all his vital signs were normal. The abdomen was distended without tenderness, and there was a giant upper abdominal mass measuring approximately 25 × 25 cm in the epigastric region, extending to both the hypochondrial areas and inferior to the level of the umbilicus. Laboratory investigations showed leukocytosis and anaemia, with a normal coagulation profile, liver, and kidney function tests (Table 1). Peripheral blood smear showed several reactive lymphocytes and a few neutrophils with toxic granulation and vacuolation. Tumour marker investigation indicated high levels of cancer antigen 125 with normal levels of alpha-1-fetoprotein, carcinoembryonic antigen, and carbohydrate antigen 19-9 (Table 2).

**Table 1:** Laboratory investigation

Investigation	Results	Normal ranges
White blood cells	14.4 × 10^9^/L	5.0–10.0 × 10^9^/L
Neutrophils absolute	5.9 × 10^9^/L	1.8–7.7 × 10^9^/L
Lymphocytes absolute	2.7 × 10^9^/L	1–3 × 10^9^/L
Monocytes absolute	0.7 × 10^9^/L	0.2–1.0 × 10^9^/L
Eosinophils absolute	0.9 × 10^9^/L	0.02–0.5 × 10^9^/L
Basophils absolute	0.08 × 10^9^/L	0.02–0.1 × 10^9^/L
Red blood cell count	4.5 × 10^6^/µL	4.5–5.5 × 10^9^/L
Haemoglobin concentration	11.8 g/dL	13–17 g/dL
Haematocrit	35.3%	40–50%
Mean cell volume	89.0 fL	83–101 fL
Mean cell haemoglobin	29.7 pg	27–33 pg
Mean corpuscular haemoglobin concentration	33.4%	31.5–34.5%
Platelet count	408 × 10^9^/L	150–410 × 10^9^/L
Red cell distribution width – coefficient of variation	13.8%	11.6–14%
Mean platelet volume	9.8 fL	9.7–12.0 fL
Activated partial thromboplastin clotting time	25.9 seconds	24–36 seconds
Prothrombin time	11.9 seconds	10.5–13.8 seconds
Partial thromboplastin time	26 seconds	25–35 seconds
International normalised ratio	0.99%	0.9–1.3%
Alanine transaminase	6 U/L	5–56 U/L
Aspartate aminotransferase	13 U/L	0–40 U/L
Alkaline phosphatase	50 U/L	44–147 U/L
Gamma-glutamyl transferase	25 U/L	5–51 U/L
Total bilirubin	8.1 µmol/L	0–21 µmol/L
Direct bilirubin	3.1 µmol/L	0–5 µmol/L
Calcium	2.38 mmol/L	2.12–2.52 mmol/L
Magnesium	0.78 mmol/L	0.74–0.99 mmol/L
Phosphorous	1.39 mmol/L	0.87–1.45 mmol/L
Potassium	4.3 mmol/L	3.5–5.3 mmol/L
Sodium	142 mmol/L	135–145 mmol/L
Creatine kinase	68 U/L	39–308 U/L
Creatinine	79 µmol/L	44–116 µmol/L
Urea serum	5.2 mmol/L	2.76–8.07 mmol/L

**Table 2:** The tumour marker investigation

Investigation	Results	Normal ranges
Cancer antigen 125	240 U/mL	0–35 U/mL
Alpha-1-fetoprotein	2.87 IU/ml	≤8.0 ng/mL
Carcinoembryonic antigen	1.24 ng/mL	0–3.0 ng/mL
Carbohydrate antigen 19-9	Less than 2 U/mL	<37 U/mL

**Figure 1:**
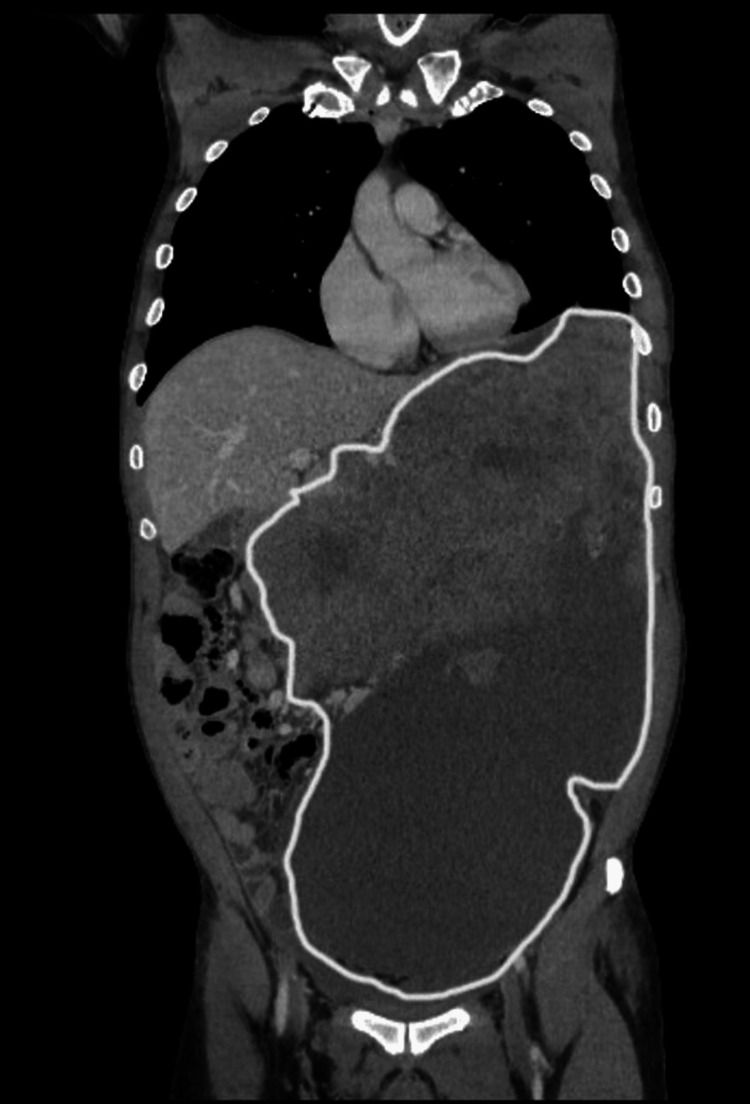
Abdominal CT images before neoadjuvant therapy with imatinib show an intraabdominal mass occupying most of the abdominal pelvic cavity, causing a significant mass effect. The mass shifts the bowel loops to the right side of the abdomen, compresses the retroperitoneal structures, and pushes the stomach inferiorly. It measures 35 x 25 x 20 cm and originates from the fundus of the stomach.

A computed tomography (CT) scan demonstrated a giant intra-abdominal mass occupying most of the abdominal pelvic cavity, which caused a significant mass effect, shifting the bowel loops to the right side of the abdomen, compressing the retroperitoneal structures, and pushing the stomach inferiorly (Figure 1). The mass measured 35 × 25 × 20 cm and likely originated from the fundus of the stomach, with many vessels supplying it, and had peri-oesophageal and peri-gastric collaterals. The mass was heterogeneous in enhancement, with cystic and necrotic components; however, there was no calcification or metastasis. Ultrasound-guided biopsy showed a GIST. Immunohistochemistry, with appropriate controls, revealed positive CD117, DOG1, and CD34; negative DESMIN; and elevated KI67. Upper gastrointestinal endoscopy exhibited external compression of the stomach and fundal varices, and a large fundal submucosal lesion measuring 3 × 3 cm. There was a severe danger of both the tumour rupturing during the surgery and the combined excision of adjacent organs if the surgery was performed with the massive tumour. The patient was informed regarding the potential risks and benefits of immediate tumour resection (surgery) and surgery after neoadjuvant therapy. The patient opted for neoadjuvant therapy and then tumour resection (surgery). Therefore, he was administered neoadjuvant therapy with imatinib 400 mg orally once daily for three months, with re-evaluation and follow-up every month. The patient was aware of his disease status and the side effects of the plan of care.

**Figure 2:**
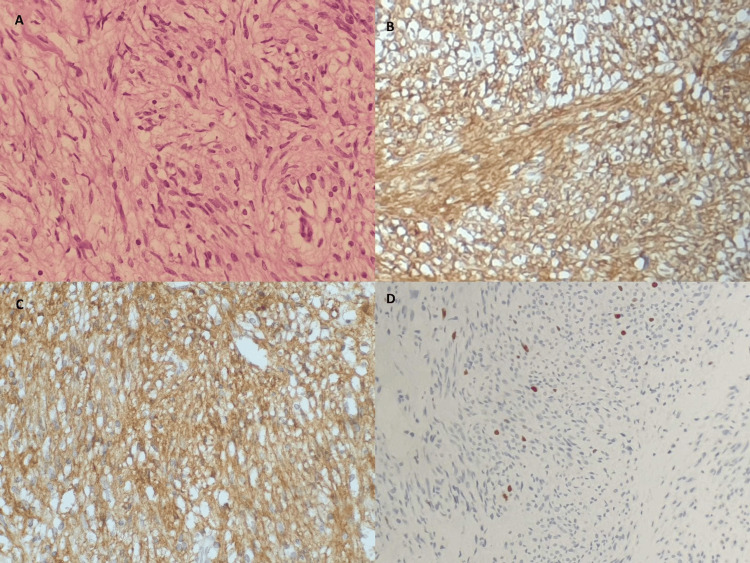
Histopathological examination after the neoadjuvant therapy. A: Hematoxylin-eosin staining showing spindle-shaped cells. B-D: Immunochemical staining was positive for CD117 (B), DOG1 (C), and KI 67 (D).

**Figure 3:**
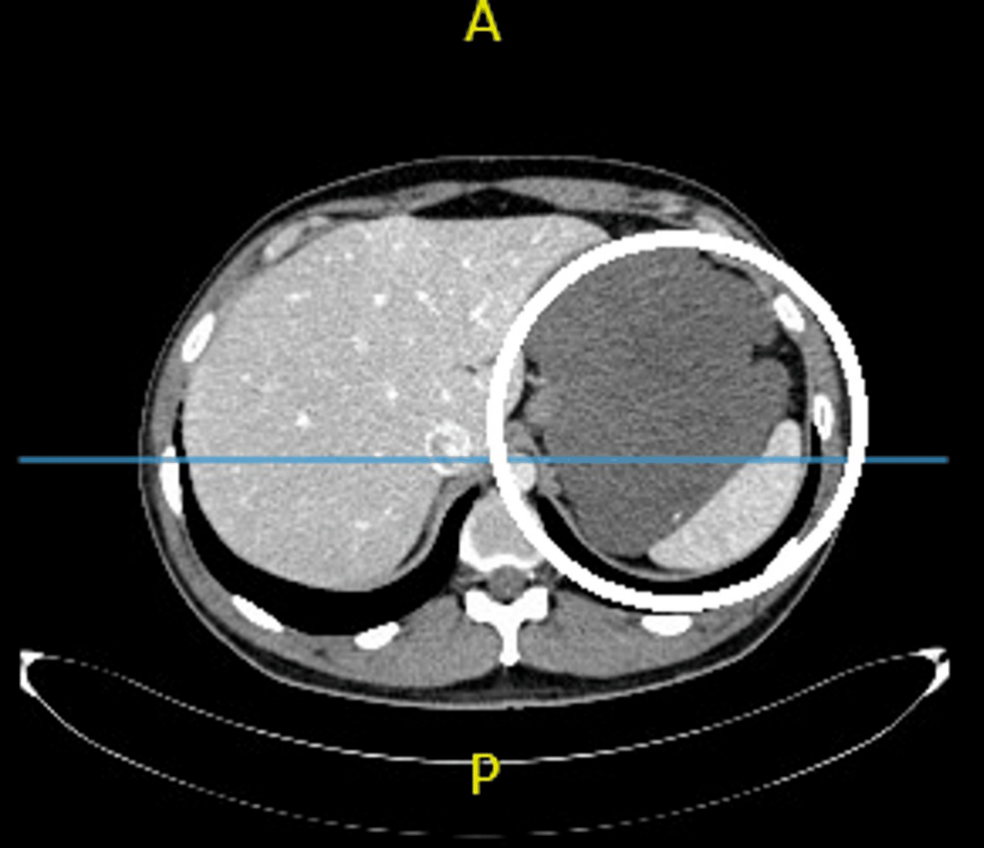
Abdominal CT images after neoadjuvant therapy with imatinib demonstrating a significant size regression and decreased enhancement of the giant intra-abdominal necrotic mass. The mass now measured 17 × 14 × 21 cm.

**Figure 4:**
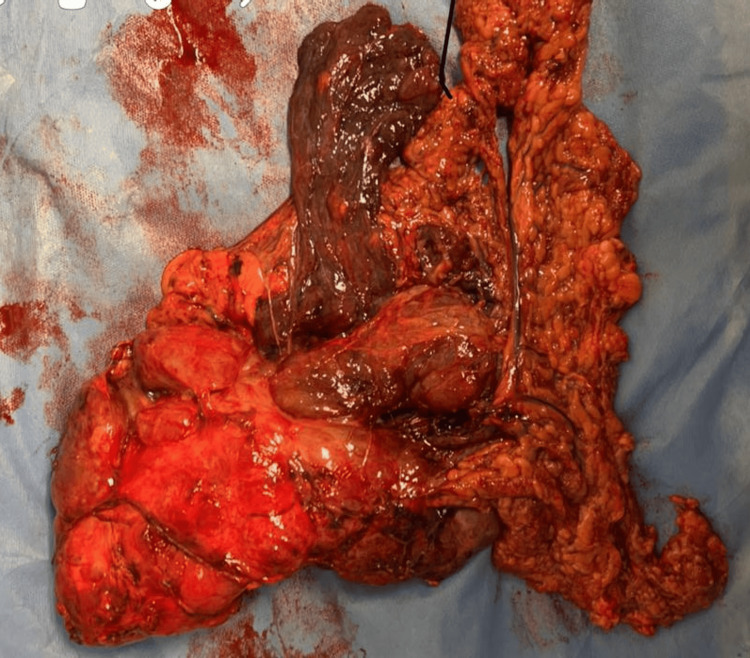
Gross view of the resected tumour.

All follow-up visits and laboratory tests were normal. During the clinical visit after three months of neoadjuvant therapy, his vital signs were normal. Histopathological examination after the neoadjuvant therapy revealed a low-grade GIST with hypocellularity, myxoid stroma, and fibrosis (Figure 2). CT demonstrated a significant size regression and decreased enhancement of the giant intra-abdominal necrotic mass, which was predominantly cystic with septations (Figure 3). The mass now measured 17 × 14 × 21 cm. The patient underwent surgical resection a month after the completion of neoadjuvant therapy. Complete resection was performed, with partial gastrectomy; the patient did not require total gastrectomy or a combined resection of other organs (Figure 4). The patient’s post-operative period was uneventful, and he was regularly followed up at the general surgery department for 24 months without recurrence.

## Discussion

We encountered an uncommon case of a 45-year-old man with a very large GIST that responded well to neoadjuvant therapy with imatinib 400 mg daily, resulting in the shrinkage of the tumour and subsequent safe and complete resection. Comprehensive surgical resection is the typical treatment for local GISTs. However, 40%-90% of patients experience relapse. Specifically, giant tumours have a risk of rupture during surgery or a positive margin despite a macroscopically comprehensive resection. These two factors, i.e., tumour rupture and the presence of a residual tumour, are significantly associated with recurrence and poor prognosis [12]. Hence, studies recommend neoadjuvant therapy for large GISTs to cause tumour shrinkage, thereby reducing the risks of tumour rupture and incomplete resection [13], and improving R2 resection rates associated with favourable survival [14]. GIST is categorized as small (<5 cm), medium (5-10 cm), and large (>10 cm), with an average size of 8.78 cm (ranging from 0.6 cm to 25.5 cm) [15,16].

A phase II study (2023) was conducted on neoadjuvant imatinib therapy for large GISTs measuring ≥10 cm, which involved 56 Asian multinational patients treated with imatinib 400 mg daily for six to nine months pre-operatively and for at least one year and preferably for three years post-operatively. The findings showed a five-year overall survival of 94.3% and progression-free survival of 61.6% [17]. A systemic review study on the efficacy of neoadjuvant, adjuvant, and lifelong medical oncological treatment for GIST (2022) demonstrated that in all reported studies, the patients received both neoadjuvant and adjuvant therapy. Hence, the effect of neoadjuvant therapy could not be distinguished from that of adjuvant therapy [18]. Neither of these studies included GISTs >30 cm, but the present case showed a good response to the neoadjuvant imatinib after three months of therapy. In a guideline, adjuvant treatment with imatinib 400 mg daily has been documented to improve relapse-free survival and recommended following complete resection for GIST with no neoadjuvant treatment. Additionally, neoadjuvant treatment with imatinib should be considered only if surgical morbidity could be reduced by downstaging the tumour before surgery [19]. The optimal duration of neoadjuvant imatinib therapy is not clear and remains controversial. The general agreement is that imatinib should be continued until the maximal response is achieved, which is defined as a lack of change in the size of the tumour between two successive CT scans [19,20]. Recent guidelines estimate that surgery could be performed between 6 and 12 months after the initiation of imatinib [19]. The main limitation of this neoadjuvant therapy is the development of secondary resistance associated with additional KIT mutations [20]. Recent data seem to confirm the presence of an inter-individual difference in the treatment response. While KIT exon 11 and 13 mutations are associated with a good response, KIT exon nine mutations and PDGFRA gene mutations are linked to a poor response [13]. Unfortunately, our patient’s mutation was not known, as genetic testing is unavailable in our hospital. The initial imatinib dose of 400 mg daily is practically considered the standard dose [19]. High doses, such as 600 and 800 mg/day as the initial dose, have also been examined in randomized and phase II trial studies. However, no obvious advantages were observed compared with 400 mg/day [21]. In the present case, three months of neoadjuvant therapy was administered with imatinib 400 mg orally once daily.

Only five case reports have been published on tumours >30 cm (Table 3), and only two of these were treated with neoadjuvant [22,23]. To the best of our knowledge, this is the third case of a giant GIST with a size of >30 cm that underwent neoadjuvant therapy. Several factors are to be considered when managing a giant gastric GIST with neoadjuvant therapy, including malignant predictors, tumour size and position, and the patient’s age and health condition. Our case supports neoadjuvant treatment as an effective strategy for managing giant gastric GISTs, especially those >30 cm, to reduce tumour size before surgery, thus lowering the surgical risk. According to guidelines, if a complex surgical procedure is required, then a multidisciplinary consultation regarding the use of neoadjuvant imatinib therapy is recommended [19].

**Table 3:** A summary of published literature on gastrointestinal stromal tumours larger than 30 cm

Number	Author/citation	Year of publication	Age	Gender	Neoadjuvant therapy	Operation	Prognosis
1	Cruz et al. [24]	2008	37	Male	No	Subtotal gastrectomy	Alive without recurrence at 12 months
2	Cappellani et al. [25]	2013	67	Male	No	Sleeve resection of stomach, distal pancreatectomy - splenectomy	Alive without recurrence at 24 months
3	Koyuncuer et al. [26]	2015	43	Male	No	Partial gastrectomy	Not mentioned
4	Takahashi et al. [22]	2020	71	Female	Yes	Partial gastrectomy	Alive without recurrence at 12 months
5	Kwon et al. [23]	2022	70	Male	Yes	Partial gastrectomy	Not mentioned
6	This study	2023	45	Male	Yes	Partial gastrectomy	Alive without recurrence at 24 months

## Conclusions

Gastric GIST is a rare disease, and its size ranges from 0.6 cm to 25.5 cm. There is a great danger of both the tumour rupturing and the need for major resection, including total gastrectomy and the resection of adjacent structures such as the colon, spleen, or small bowel, if the surgery is performed immediately on a giant tumour. Therefore, we recommend that neoadjuvant therapy be considered in patients with large GISTs or local invasion of other structures to reduce the tumour size before surgery. This approach enhances the complete resection rate, prevents the need for multi-organ resection, and lowers the risk of surgery.
